# Serologic Intestinal-Fatty Acid Binding Protein in Necrotizing Enterocolitis Diagnosis: A Meta-Analysis

**DOI:** 10.1155/2015/156704

**Published:** 2015-12-22

**Authors:** Shupeng Cheng, Jialin Yu, Min Zhou, Yan Tu, Qi Lu

**Affiliations:** Department of Neonatology, Children's Hospital of Chongqing Medical University, Ministry of Education Key Laboratory of Child Development and Disorders, Chongqing 400014, China

## Abstract

*Background*. Previous studies showed that intestinal-fatty acid binding protein (I-FABP) may be a valid and promising serologic biomarker for early diagnosis of necrotizing enterocolitis (NEC).* Objective*. To investigate the early diagnostic value of serologic I-FABP in NEC for the premature neonates.* Methods*. All major databases were searched from January 1, 1990, to May 1, 2015. We used Meta-Disc 1.4 and Revman5.0 software to calculate the diagnostic accuracy.* Results*. Seven studies with 444 subjects were identified. The pooled sensitivity of I-FABP was 0.67 for NEC I, 0.74 for NEC II, and 0.83 for NEC III, and the pooled specificity was 0.84, respectively, which showed a moderate diagnostic accuracy. The area under curve (AUC) for each stage was 0.75 (*Q* ^⁎^ = 0.69), 0.82 (*Q* ^⁎^ = 0.76), and 0.91 (*Q* ^⁎^ = 0.84). The diagnostic threshold analysis showed no significant difference in threshold effect. The metaregression showed that the cut-off value has the largest effect on heterogeneity. The funnel plots indicated the existence of publication bias.* Conclusion*. I-FABP is a valid serologic biomarker for early diagnosis in NEC for the premature neonates with a moderate accuracy.

## 1. Introduction

Necrotizing enterocolitis (NEC) is a common and devastating condition in neonates and remains a leading cause of morbidity and mortality (20–40%), especially for the preterm infants [1].

Diagnosis of NEC was made by clinical and radiological signs according to modified Bell's staging criteria at present [2]. However, relying on clinical manifestations tends to heighten omission diagnostic rate because both systemic and abdominal signs are nonspecific in NICU patients. These signs include feeding intolerance, abdominal distention, bloody stool, dyspepsia, and ascites [3]. Similarly, the radiological signs often lack sufficient discriminative power due to time delay [4]. Accurate and timely diagnosis will limit morbidity, improve patients' living quality, and reduce costs. Therefore, we urgently need a new diagnostic method that is valid and promising for early diagnosis.

Serologic intestinal-fatty acid binding protein (I-FABP), a small (14-15 kDa) water-soluble protein, has been studied as an early diagnostic biomarker for NEC in the past decade. This small cytosolic protein, located mainly in enterocytes of the small intestine, is released into the blood stream after intestinal ischemia and cell disruption [5]. Several studies have suggested the level of I-FABP could be used as a promising biomarker for early diagnosis and the prediction of severe NEC, even possibly for timing of surgery [6, 7]. Currently, serologic I-FABP has been proved to be available in some medical institutions, but the diagnostic efficacy still remains controversial. For example, multicenter studies with large sample size and systematic review on I-FABP for early diagnosis are lacking. So, we conducted this meta-analysis to systematically evaluate the validity of I-FABP for early diagnosis in NEC.

## 2. Methods

### 2.1. Data Source

Web of Science, Embase, Medline databases, Cochrane Library, CNKI, VIP, and other Chinese medical databases were searched from January 1, 1990, to May 1, 2015. The search terms were “necrotizing enterocolitis”, “NEC”, “newborn”, “neonate”, “biomarker”, “I-FABP”, and “intestinal-fatty acid binding protein”. The reference lists from original articles were also examined, and we contacted authors to obtain further information by email, if necessary.

### 2.2. Inclusion and Exclusion Criteria

Studies that met the following eligibility criteria were included: (1) studies that assessed the diagnostic accuracy of serologic I-FABP in NEC; (2) studies that included case group (neonates who were evaluated with suspected NEC of Bell stage ≥ *I*) [2, 3] and control group (gestational age and weight-matched neonates who were admitted to the same institution without NEC, sepsis, or systemic inflammatory response syndrome); (3) studies that provided sufficient information to construct the two-by-two tables; (4) studies that only contained neonates (within 28 days after birth); (5) studies that had well-defined reference standard and staging criteria for NEC.

Studies were excluded for the following reasons: (1) a letter, case report, and comment and (2) neonates with other gastrointestinal diseases, immunodeficiency disease, inheritance metabolism disorders, and severe congenital malformation. If studies had overlapping subjects, only the most recent information or the largest sample size of patients was included. Articles were independently reviewed by two evaluators, and any disagreements were resolved by consensus.

### 2.3. Data Extraction and Quality Assessment

Two reviewers independently extracted data from all eligible studies with a predefined information sheet which included country, year of publication, clinical setting, demographics, type of study, sample size, cut-off points, test methods, and true-positive, false-negative, false-positive, and true-negative value. If we needed any additional information that was not reported in the published articles, we requested it through electronic communication with the corresponding authors of the studies. If no reply was received, the studies were excluded from the meta-analysis.

The methodological quality of each included study was assessed based on the quality assessment with diagnostic accuracy (QUADAS) tool including 11 key items. Each item with “yes,” “no,” and “unclear” answer was scored as 1, −1, and 0, respectively [8].

### 2.4. Statistical Analysis

All statistical analyses were carried out using Revman5.0 and Meta-Disc 1.4 software for Windows [9]. We calculated the following measures of each study: sensitivity, specificity, diagnostic odds ratios (DOR), positive likelihood ratio (PLR), and negative likelihood ratio (NLR) with corresponding 95% confidence intervals, specific to different stages (NEC I, NEC II, and NEC III) [10–13]. To detect heterogeneity, the diagnostic odds ratio (DOR) was graphically displayed using forest plot and analysed using Cochran-*Q* test *I* ^2^ test. The DOR compares the odds of true-positive patients (=sensitivity) with that of false-positives (=1 − specificity) and thus summarizes the overall accuracy of a diagnostic test. A *p* value of less than 0.05 or *I* ^2^ greater than 50% indicated significant heterogeneity [14]. Fixed-effects model was used if the result of the *Q* test was not significant; otherwise, the random-effects model was used [15]. Then, a summary receiver operator characteristic (SROC) curve was used to summarize these results among all studies and the area under SROC was also calculated to show the diagnostic accuracy. *Q* ^*∗*^ point on the SROC curve was used to obtain the maximum joint sensitivity and specificity [16]. The metaregression analysis was conducted to investigate the confounding factors for heterogeneity, such as cut-off value, study quality (QUADAS), and testing time of plasma samples [17].

Publication bias is common and inevitable in a meta-analysis [18]. As we know, article with a positive result is more likely to be published, following the issue of overestimating the diagnostic performance of serologic I-FABP. In order to solve the problem, we searched different databases for more articles. Besides, publication bias was examined visually by funnel plot, and an asymmetric plot suggested possible publication bias. *p* < 0.05 was considered significant.

## 3. Results

### 3.1. Study Characteristics and Quality Assessment

**Figure 1:**
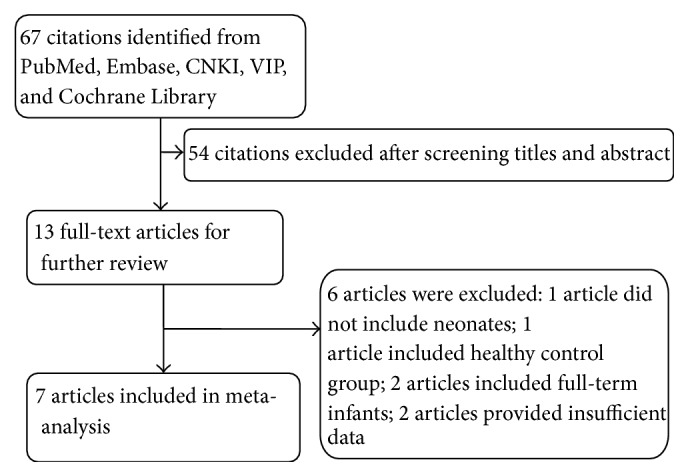
Flow diagram highlighting the process of identification and inclusion of studies in the meta-analysis.

**Figure 2:**
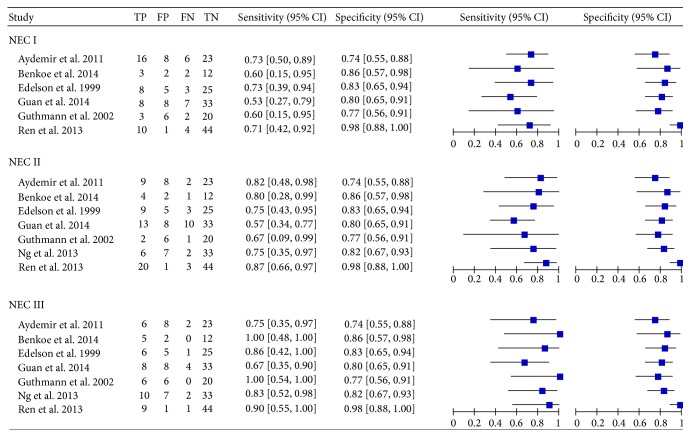
Forest plot for sensitivity and specificity of I-FABP in NEC diagnosis. TP: true-positive; TN: true-negative; FP: false-positive; FN: false-negative; NEC: necrotizing enterocolitis; I-FABP: intestinal-fatty acid binding protein; CI: confidence interval.

**Figure 3:**
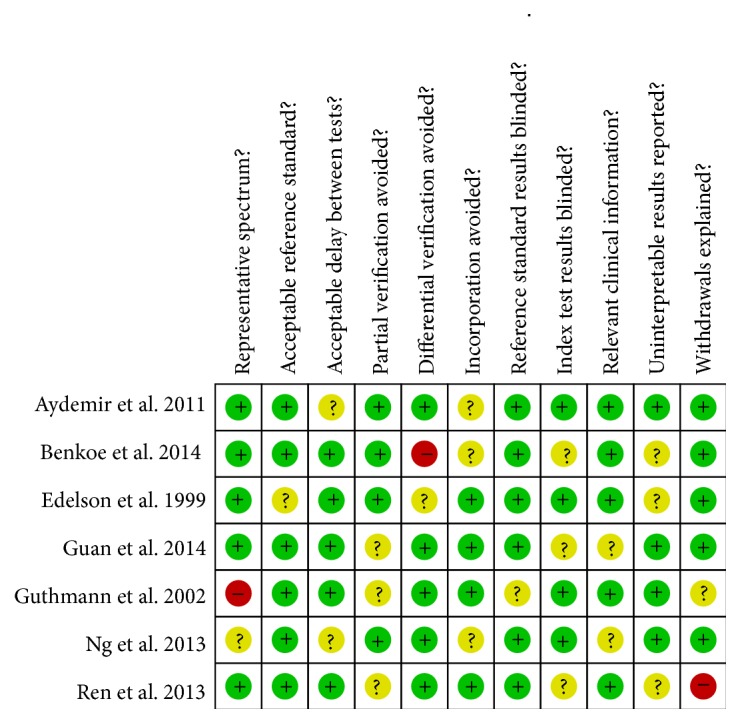
QUADAS results about the level of risk of bias for included studies.

The literature search was carried out as described, and 13 studies were considered potentially suitable. After full-text review, 4 studies were excluded: one included healthy neonates as controls, two provided insufficient information, and one included patients who were not neonates. In addition, two studies on term neonates were also excluded because of insufficient data. One article with a disagreement between both evaluators was resolved with consensus. Finally, seven publications with 444 neonates met the inclusion criteria and were admitted in the meta-analysis [19–24, 26].  Figure 1 shows the selecting process of studies. The sensitivity, specificity, and true-positive (TP), false-positive (FP), true-negative (TN), and false-negative (FN) value of each article were shown in Figure 2. The cut-off values ranged from 0.76 ng/mL to 7.70 ng/mL. More detailed characteristics of each included study were presented in Table 1. All the conditions and methods used for QUADAS of included studies were shown in Figure 3.

### 3.2. Diagnostic Accuracy

A random-effects model was used to assess the pooled value of serologic I-FABP in NEC diagnosis because of the potential heterogeneity caused by nonthreshold effect. For each stage, the sensitivity (0.67 [0.55–0.77], 0.74 [0.63–0.83], and 0.83 [0.71–0.92]), specificity (0.84 [0.78–0.89]), and DOR (10.42 [2.84–38.28], 15.82 [4.37–57.19], and 21.26 [6.53–69.21]) were performed by forest plots (Figure 2), and the PLR and NLR were showed in Table 2. All the results suggested a moderate accuracy of I-FABP for early diagnosis in NEC.

### 3.3. Analysis of Heterogeneity

**Figure 4:**
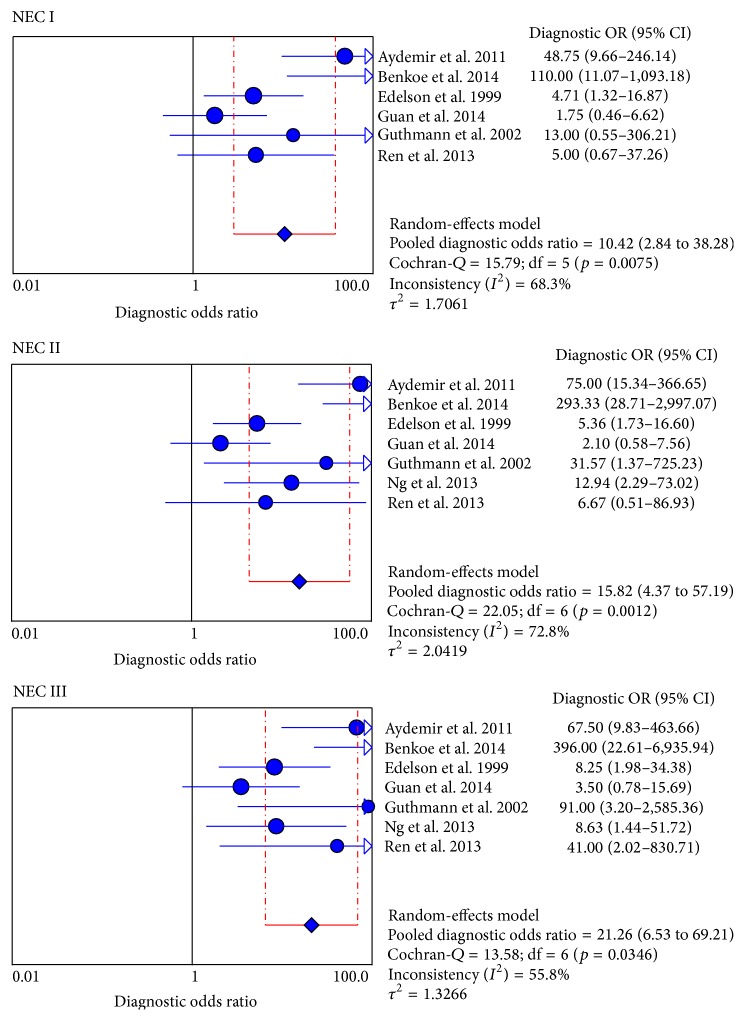
Forest plot for DOR of I-FABP in NEC diagnosis. DOR: diagnostic odds ratios; NEC: necrotizing enterocolitis; I-FABP: intestinal-fatty acid binding protein; CI: confidence interval.

Heterogeneity can signally influence the diagnostic accuracy of a meta-analysis. In this meta-analysis, heterogeneity that was explored was cut-off values, study quality (QUADAS), and testing time of plasma samples. First, we explored the threshold effect with calculating the Spearman correlation coefficient with Moses' model weighted by inverse variance. The results showed no threshold effect (*p*_I_ = 0.53, *p*_II_ = 0.48, and *p*_III_ = 0.61). Then, we used the forest plot of diagnostic odds ratios to assess the nonthreshold effect with random-effects model, and Cochran-*Q* value suggested the nonthreshold effect was statistically significant for each stage (*p*_I_ = 0.01, *p*_II_ = 0.00, and *p*_III_ = 0.03) (Figure 4, Table 2).

The reasons for heterogeneity were explored by metaregression analysis with Meta-Disc 1.4 software. The process was turning the variations such as cut-off value, study quality (QUADAS), and testing time of plasma samples from left “covariates” to the right “model” and then removes the covariate and analyzes accordingly the descending *p* values, respectively. The results showed that cut-off value was the main factor for heterogeneity (RDOR = 4.41, *p* value = 0.0650) in Table 3.

### 3.4. Analysis of SROC

**Figure 5:**
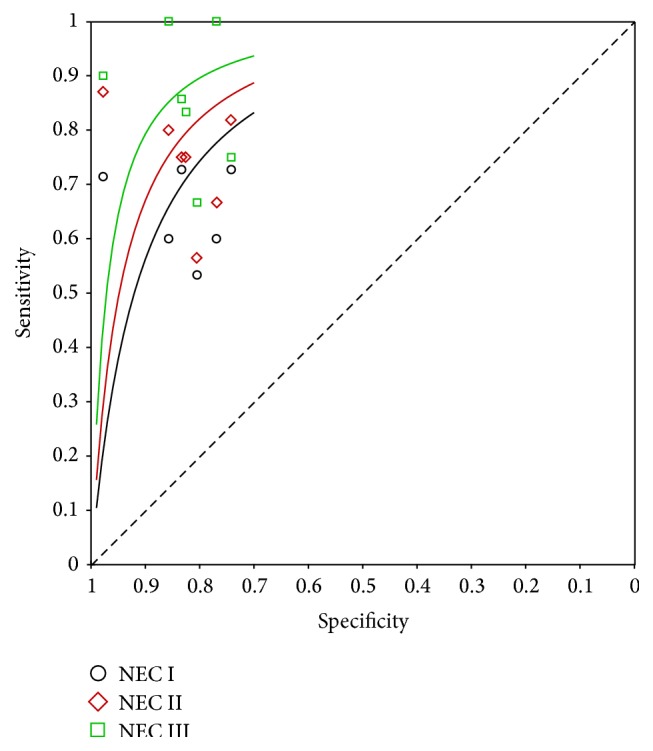
Summary receiver operating characteristic (SROC) curve of the I-FABP test for the diagnosis of NEC I, NEC II, and NEC III stages. I-FABP: intestinal-fatty acid binding protein; NEC: necrotizing enterocolitis; SROC: summary receiver operating characteristic.

We found that the summary receiver operating characteristic (SROC) curve was positioned near the upper left corner of the curve. The maximum joint sensitivity and specificity (*Q* ^*∗*^ value) was 0.69 for NEC I, 0.76 for NEC II, and 0.84 for NEC III, and the area under curve (AUC) was 0.75, 0.82, and 0.91 for each stage, consistent with a moderate diagnostic accuracy of I-FABP for early diagnosis in NEC (Figure 5, Table 2).

### 3.5. Publication Bias

**Figure 6:**
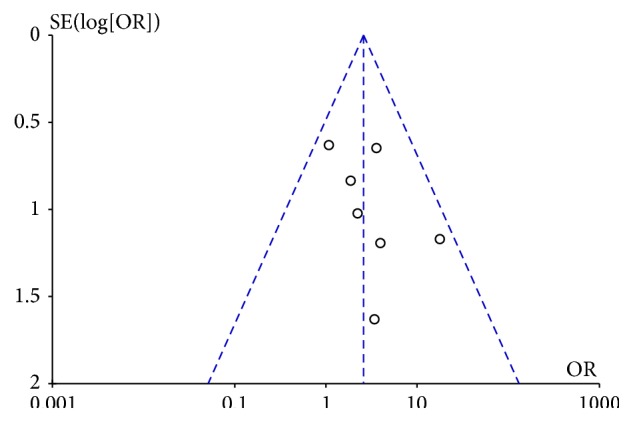
Funnel plot to estimate the publication bias of the meta-analysis.

The visual funnel plot was asymmetric, showing a potential publication bias among studies. Considering the similarity between the three funnel plots for each stage, we just selected one at random (Figure 6).

## 4. Discussion

Necrotizing enterocolitis is one of the most severe diseases and an important cause of mortality and morbidity in neonates. Early symptoms and clinical signs are nonspecific, and the radiological signs often lack sufficient discriminative power. Therefore, it is necessary for us to study valid markers for future research of NEC.

In this meta-analysis, we can find that the area under curve (AUC) was 0.75, 0.82, and 0.91 and *Q* ^*∗*^ value in SROC curve was 0.69, 0.76, and 0.84 for each stage, indicating a moderate pooled accuracy of I-FABP in diagnosing NEC. Similarly, the pooled values of sensitivity (0.67 [0.55–0.77], 0.74 [0.63–0.83], and 0.83 [0.71–0.92]) and specificity (0.84 [0.78–0.89]) suggested the potential diagnostic value of I-FABP for early detection, and DOR (10.42 versus 15.82 versus 21.26) also showed a moderate diagnostic accuracy for diagnosing NEC.

Serologic I-FABP is a specific biomarker and is convenient to detect, which is primarily located in enterocytes of the small intestine and released into the circulation after NEC [5]. Other markers include C-reactive protein, an acute phase protein that becomes rapidly elevated with a series of infectious and inflammatory conditions. Several articles have proved CRP is a relatively sensitive (91%) but nonspecific marker (65%) for NEC early diagnosis [27, 28]. Like CRP, Trefoil factor 3 (TFF3) is also a sensitive (85%) but nonspecific marker (59%) for NEC [24]. Cytokines like IL-6 and IL-8 were thought to be reliable indicators; however, there were insufficient data available from individual studies assessing cytokines in diagnosing NEC to present pooled estimates of diagnostic accuracy [27]. Reisinger et al. reported the combination of urinary Serum Amyloid A (SAA) with platelet count is an accurate detection method in diagnosing severe NEC, with higher sensitivity (94%), specificity (83%), and AUC area (0.95) [29]. Benkoe et al. reported a high diagnostic accuracy and clinically relevant value of fecal calprotectin (AUC = 0.94) for diagnosing NEC, with a limitation in using the stool samples which cannot be obtained in some patients with NEC or non-NEC [20]. In addition, Aydemir et al. reported fecal calprotectin is a useful marker in differentiating severe NEC from early NEC with 76% sensitivity and 92% specificity [30].

An exploration of the source for heterogeneity rather than a summary computation was an important goal of this meta-analysis. Metaregression has proved that the cut-off value was the main factor, which may partially explain the heterogeneity because of the differences between studies. Hence, we expect to reduce the heterogeneity by including more studies for I-FABP test in the future.

The meta-analysis showed that serologic I-FABP was a helpful biomarker for early diagnosis of NEC for the premature neonates. Additionally, the study population in this review just included preterm neonates, and we supposed our findings may be also appropriate for early identifying full-term neonates with NEC, though the disease process in these two populations is markedly different [31]. However, few studies about full-term neonates could be searched to validate our supposition.

Although our results are valuable and promising, several limitations still existed in our study. First, the language barrier and limited studies might have led to some bias. In general, a large sample size can diminish any bias and make the conclusion more convincing. Second, some differences in the used ELISA's kits may also influence the results.

## 5. Conclusion

In summary, I-FABP is a valid serologic biomarker for early diagnosis of NEC for the premature neonates with a moderate accuracy; thus it may serve as a new auxiliary diagnosis method and decrease the omission diagnostic rate. To provide a more reliable diagnostic basis for clinical implement, further large scale and multicenter prospective studies are needed.

## Figures and Tables

**Table 1:** Characteristics of the studies examining the role of I-FABP in NEC diagnosis.

Study	Country	Population (*n*)			Control	Demographic characteristic		Testing time^1^	Measure method	Cut-off (ng/mL)	QUADAS
		NEC I	NEC II	NEC III		Birth weight (g)	Gestational age (wk)				
Aydemir et al. 2011 [19]	Turkey	22	11	8	31	Cases: 1410 ± 315 g Controls: 1454 ± 276 g	Cases: 30.2 ± 1.2 wk Control: 30.4 ± 1.3 wk	At onset and 24 and 72 h point	ELISA	0.76	7

Benkoe et al. 2014 [20]	Austria	5	5	5	14	Cases: 1156 ± 507 g Controls: 1128 ± 702 g	Cases: 29.0 ± 0.6 wk Control: 28.6 ± 1.1 wk	At onset, 24 h point	RIA	2.80	6

Edelson et al. 1999 [21]	America	11	12	7	30	Cases: 1251 ± 119 g Controls: 1277 ± 271 g	Cases: 28.9 ± 1.2 wk Control: 29.1 ± 0.6 wk	<24 h	ELISA	1.87	8

Guan et al. 2014 [22]	China	15	23	12	41	Cases: 1211 ± 315 g Controls: 1198 ± 691 g	Cases: 28.1 ± 1.5 wk Control: 27.9 ± 1.1 wk	At 8, 16, and 24 h	ELISA	2.00	8

Guthmann et al. 2002 [23]	Germany	5	3	6	26	Cases: 1298 ± 572 g Controls: 1342 ± 180 g	Cases: 29.8 ± 0.2 wk Control: 30.4 ± 1.7 wk	<24 h	ELISA	2.52	7

Ng et al. 2013 [24]	Hong Kong	0	8	12	40	Cases: 1143 ± 267 g Controls: 1113 ± 383 g	Cases: 28.4 ± 1.8 wk Control: 27.8 ± 1.6 wk	At 24 and 72 h	ELISA	7.70	7

Ren et al. 2013 [25]	China	14	23	10	45	Cases: 1220 ± 103 g Controls: 1290 ± 125 g	Cases: 29.1 ± 0.9 wk Control: 30.4 ± 1.1 wk	At onset and 8, 24, and 72 h point	ELISA	3.20	5

^1^Testing time: testing time of plasma sample.

**Table 2:** PLR, NLR, DOR, AUC, and *Q* ^*∗*^ value for each stage.

Stage	PLR (95% CI)	NLR (95% CI)	DOR (95% CI)	*p*	AUC	*Q* ^*∗*^
NEC I	3.54 (2.29–5.46)	0.45 (0.33–0.60)	10.42 (2.84–38.28)	0.01	0.75	0.69
NEC II	4.23 (2.49–7.18)	0.33 (0.21–0.51)	15.82 (4.37–57.19)	0.00	0.82	0.76
NEC III	4.49 (2.85–7.09)	0.25 (0.15–0.43)	21.26 (6.53–69.21)	0.03	0.91	0.84

PLR: positive likelihood ratio; NLR: negative likelihood ratio; DOR: diagnostic odds ratios; AUC: area under curve; CI: confidence interval; *Q* ^*∗*^: the maximum joint sensitivity and specificity.

**(a):** 

Covariates	Coefficient	Stand. error	RDOR (95% CI)	*p* value
Cut-off	0.508	0.3570	4.41 (1.25; 16.47)	0.0650
QUADAS	0.899	0.9412	1.53 (0.19; 11.82)	0.7290
Testing time	1.319	1.3255	2.36 (0.69; 21.44)	0.2862

**(b):** 

Covariates	Coefficient	Stand. error	RDOR (95% CI)	*p* value
Cut-off	0.659	0.5387	5.16 (1.87; 14.11)	0.1847
Testing time	1.587	1.8118	2.35 (0.58; 23.35)	0.4376

**(c):** 

Covariates	Coefficient	Stand. error	RDOR (95% CI)	*p* value

Cut-off	0.715	0.6575	4.98 (1.64; 14.92)	0.2713
